# Graph theoretical analysis of developmental patterns of the white matter network

**DOI:** 10.3389/fnhum.2013.00716

**Published:** 2013-11-01

**Authors:** Zhang Chen, Min Liu, Donald W. Gross, Christian Beaulieu

**Affiliations:** ^1^Department of Biomedical Engineering, Faculty of Medicine and Dentistry, University of AlbertaEdmonton, AB, Canada; ^2^Division of Neurology, Department of Medicine, Faculty of Medicine and Dentistry, University of AlbertaEdmonton, AB, Canada

**Keywords:** graph theory, neurodevelopment, anatomical connectivity, modular networks, small world network

## Abstract

Understanding the development of human brain organization is critical for gaining insight into how the enhancement of cognitive processes is related to the fine-tuning of the brain network. However, the developmental trajectory of the large-scale white matter (WM) network is not fully understood. Here, using graph theory, we examine developmental changes in the organization of WM networks in 180 typically-developing participants. WM networks were constructed using whole brain tractography and 78 cortical regions of interest were extracted from each participant. The subjects were first divided into 5 equal sample size (*n* = 36) groups (early childhood: 6.0–9.7 years; late childhood: 9.8–12.7 years; adolescence: 12.9–17.5 years; young adult: 17.6–21.8 years; adult: 21.9–29.6 years). Most prominent changes in the topological properties of developing brain networks occur at late childhood and adolescence. During late childhood period, the structural brain network showed significant increase in the global efficiency but decrease in modularity, suggesting a shift of topological organization toward a more randomized configuration. However, while preserving most topological features, there was a significant increase in the local efficiency at adolescence, suggesting the dynamic process of rewiring and rebalancing brain connections at different growth stages. In addition, several pivotal hubs were identified that are vital for the global coordination of information flow over the whole brain network across all age groups. Significant increases of nodal efficiency were present in several regions such as precuneus at late childhood. Finally, a stable and functionally/anatomically related modular organization was identified throughout the development of the WM network. This study used network analysis to elucidate the topological changes in brain maturation, paving the way for developing novel methods for analyzing disrupted brain connectivity in neurodevelopmental disorders.

## Introduction

Neuroimaging studies have demonstrated widespread and regionally specific structural and functional brain changes during development from infancy to adulthood. Structural magnetic resonance imaging (MRI) studies have reported age-related changes in brain volumes (Giedd et al., 1999; Good et al., 2001), areas (Thompson et al., 2000), cortical thickness (Sowell et al., 2004; Shaw et al., 2008), and regional gray matter (GM) and white matter (WM) density (Paus et al., 1999; Gogtay et al., 2004). The developmental changes in GM and WM on gross scale MRI may reflect synaptic pruning and myelination occurring at the neuronal level (Gogtay et al., 2004). Functional neuroimaging studies have demonstrated increased connectivity among distant regions and decreased connectivity among neighboring regions in brain maturation which suggests a mechanism of segregation of local regions and integration of distant regions into disparate subnetworks for the developing brain (Fair et al., 2008, 2009; Vogel et al., 2010). Diffusion tensor imaging (DTI) studies of WM have shown age-related increases in fractional anisotropy (FA) and decrease in overall diffusion with development [many studies but some include (Snook et al., 2005; Lebel et al., 2008; Tamnes et al., 2010)], including into young adulthood (Giorgio et al., 2008; Lebel and Beaulieu, 2011).

The recent advent of modern network analysis based on graph theory (Strogatz, 2001), has enabled the investigation of the large-scale topological organization of various structural and functional brain networks such as the small-world property, network efficiency and modularity (He et al., 2007; Bullmore and Sporns, 2009; He and Evans, 2010). The network metrics have also proven useful in modeling the large-scale functional and structural organization of the developing brain. Several functional brain network studies have reported age-related increases in the small-worldness (Wu et al., 2013) and a progression from local to distributed organization (Fair et al., 2009) in brain development. The analysis of the structural brain network constructed from regional cortical thickness correlations has revealed a non-linear developmental pattern in network metrics and that most topological changes happen at the late childhood stage (Khundrakpam et al., 2013).

Recently, there has been an increasing interest in the study of how graph metrics of the anatomical brain network change during development. Using DTI, Yap et al. (2011) examined WM networks of 39 healthy pediatric subjects with longitudinal data collected at average ages of 2 weeks, 1 year, and 2 years and demonstrated that the small-world architecture exists at birth with efficiency that increases in later stages of development. Two recent brain connectivity studies of WM maturation pattern using diffusion MRI tractography demonstrated linear and non-linear patterns of increasing structural efficiency with age between ages 2 and 18 years in 30 patients scanned clinically and otherwise deemed normal post-MRI (Hagmann et al., 2010) and between ages 12 and 30 years in 439 healthy subjects (Dennis et al., 2013). However, those studies were limited by different constraints such as a binarized brain network, limited sample size, or restricted age range (early adolescence to early adulthood), thus the developmental trajectory of the WM network from early childhood to adulthood remains unclear.

Therefore, the main goal of this study was to map the developmental changes of the structural brain network based on WM connectivity in 180 typically-developing subjects from 6 to 30 years of age. We hypothesized (i) non-linear age-related developmental trajectories of network metrics as most changes would be expected to happen at late childhood and adolescent stages, and (ii) altered modular organization in different age groups that reflects a process of fine-tuning in structural brain development.

## Materials and methods

### Subjects

This study included 180 healthy right-handed subjects aged from 6 to 30 years. Health was verified by asking participants a series of questions to ensure there was no history of neurological or psychiatric disease or brain injury. All subjects gave informed consent; child assent and parent/guardian consent was obtained for volunteers under 18 years. The subjects were divided into 5 age groups with equal numbers of subjects and demographics of all groups are shown in Table 1.

**Table 1:** **Group demographics**.

**Group**	**Early childhood**	**Late childhood**	**Adolescence**	**Young adult**	**Adult**
Number	36	36	36	36	36
Male/female	16/20	19/17	15/21	18/18	16/20
Mean age, *SD* (*y*)	8.1 (1.1)	11.3 (0.9)	15.4 (1.4)	19.4 (1.1)	25.7 (2.7)
Age range (*y*)	6.0–9.8	9.9–12.7	12.9–17.6	17.6–21.8	21.9–29.7

### Image acquisition

All data were acquired on a 1.5-T Siemens Sonata MRI scanner. Standard DTI was acquired using a dual spin-echo, single shot echo-planar imaging sequence with the following parameters: 40 3-mm-thick slices with no inter-slice gap, *TR* = 6400 ms, *TE* = 88 ms, 6 non-collinear diffusion sensitizing gradient directions with *b* = 1000 s/mm^2^, 8 averages, field-of-view 220 × 220 mm^2^, matrix of 96 × 128 zero-filled to 256 × 256, and scan time of 6:06 min. T1-weighted images were also acquired using MPRAGE with *TE* = 4.38 ms, *TR* = 1870 ms, *TI* = 1100 ms, and scan time of 4:29 min.

### Structural brain network construction

**Figure 1:**
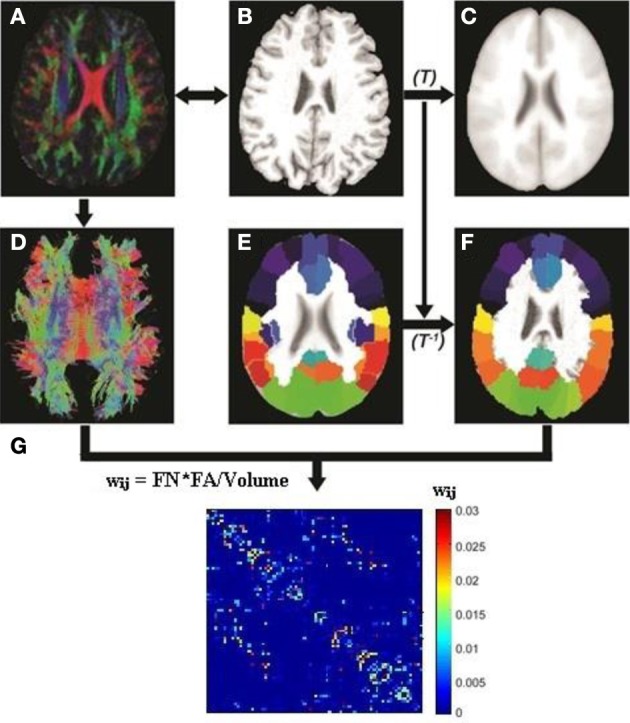
**Flowchart for the construction of the DTI white matter (WM) network for each subject**. The T_1_-weighted image of each subject **(B)** was first coregistered into DTI native space **(A)** using rigid transformation to the b0 image (not shown). The resultant T1 image was then non-linearly registered to the ICBM 152 template **(C)** in the MNI space to obtain transformation matrix *T*. The AAL template **(E)** was then inversely warped back to the individual DTI space **(F)** using the inverse transformation (*T*^−1^). Whole brain white matter fibers were reconstructed using a deterministic tractography method in native DTI space **(D)**. The WM fibers connecting any pair of regions were located and the edge weight between the two regions was calculated from the FA, fiber number (FN) and average volume of the two cortical regions. **(G)** A sample white matter network for one subject.

Image preprocessing steps including motion and eddy current corrections were performed using FSL 5.0 for all DTI images (http://fsl.fmrib.ox.ac.uk/fsl/fslwiki). The T_1_-weighted (MPRAGE) image of each subject was first linearly coregistered (Figures 1A,B) to its corresponding b0 image. Each transformed T_1_ image was then non-linearly registered to a pre-segmented and validated volumetric template, the automated anatomical labeling (AAL) atlas (Tzourio-Mazoyer et al., 2002) as shown in Figures 1B,C. This parcellation divided the cortical surface into 78 regions (39 per hemisphere). See Table 2 for the name of the regions and their corresponding abbreviations. The resulting inverse deformation map (*T*^−1^) for each subject was then applied to warp the AAL template to the DTI native space of each subject using nearest neighbor interpolation method (Figures 1E,F) as each AAL region was defined as a brain network node. Whole brain WM tractography was performed using a brute-force streamline-tracking method (Basser et al., 2000) with a FA threshold of 0.2 and primary eigenvector turning angle of 45 degrees (Figures 1A,D). To limit false positive connections, two cortical regions were deemed connected if at least 10 connecting fibers with two end points were located between them; the same threshold was also applied in a recent brain network study (van den Heuvel et al., 2012). The effects of different node-connecting fiber number (FN) thresholds ranging from 3 to 10 were determined for our network analysis. We quantified the weight of each valid connection between two cortical regions (*i* and *j*) as the product of the connecting FN and mean FA of the connecting fiber, normalized by dividing the average volume of the two connecting regions to counteract the bias where larger cortical regions inherently project/receive more “virtual” fibers (*w*_*ij*_ = FN^*^FA/Volume). Several diffusion brain network studies have applied this weighting function (Lo et al., 2010; Brown et al., 2011). As a result, the structural brain network of each participant was represented by a symmetric 78 × 78 matrix (Figure 1G).

**Table 2:** **Seventy eight cortical regions of interest defined in the study and their abbreviations (L: left hemisphere, R: right hemisphere)**.

**Index (L, R)**	**Region**	**Abb**.	**Index (L, R)**	**Regions**	**Abb**.
(1, 40)	Gyrus rectus	REC	(21, 60)	Precuneus	PCUN
(2, 41)	Olfactory cortex	OLF	(22, 61)	Superior occipital gyrus	SOG
(3, 42)	Superior frontal gyrus, orbital part	ORBsup	(23, 62)	Middle occipital gyrus	MOG
(4, 43)	Superior frontal gyrus, medial orbital	ORBsupmed	(24, 63)	Inferior occipital gyrus	IOG
(5, 44)	Middle frontal gyrus orbital part	ORBmid	(25, 64)	Calcarine fissure and surrounding cortex	CAL
(6, 45)	Inferior frontal gyrus, orbital part	ORBinf	(26, 65)	Cuneus	CUN
(7, 46)	Superior frontal gyrus, dorsolateral	SFGdor	(27, 66)	Lingual gyrus	LING
(8, 47)	Middle frontal gyrus	MFG	(28, 67)	Fusiform gyrus	FFG
(9, 48)	Inferior frontal gyrus, opercular part	IFGoperc	(29, 68)	Heschl gyrus	HES
(10, 49)	Inferior frontal gyrus, triangular part	IFGtriang	(30, 69)	Superior temporal gyrus	STG
(11, 50)	Superior frontal gyrus, medial	SFGmed	(31, 70)	Middle temporal gyrus	MTG
(12, 51)	Supplementary motor area	SMA	(32, 71)	Inferior temporal gyrus	ITG
(13, 52)	Paracentral lobule	PCL	(33, 72)	Temporal pole: superior temporal gyrus	TPOsup
(14, 53)	Precentral gyrus	PreCG	(34, 73)	Temporal pole: middle temporal gyrus	TPOmid
(15, 54)	Rolandic operculum	ROL	(35, 74)	Parahippocampal gyrus	PHG
(16, 55)	Postcentral gyrus	PoCG	(36, 75)	Anterior cingulate and paracingulate gyrus	ACG
(17, 56)	Superior parietal gyrus	SPG	(37, 76)	Median cingulate and paracingulate gyrus	DCG
(18, 57)	Inferior parietal, but supramarginal and angular gyri	IPL	(38, 77)	Posterior cingulate gyrus	PCG
(19, 58)	Supramarginal gyrus	SMG	(39, 78)	Insula	INS
(20, 59)	Angular gyrus	ANG			

To examine the small-worldness and modular organization of the WM networks for all different age groups, one weighted backbone network for each age group was generated to capture the underlying anatomical connectivity patterns using a previously published method by our group (Gong et al., 2009). In summary, to identify the highly consistent cortical connections, a non-parametric one-tailed sign test was applied. For each pair of cortical regions, the sign test was performed with the null hypothesis that there is no existing connection. The Bonferroni method was applied to correct for multiple comparisons at *P* < 0.05. The use of this conservative statistical criterion generated a symmetric weighted matrix as each edge weight was calculated as the mean of all existing edges in all subjects that captured underlying anatomical connectivity patterns in the human cerebral cortex (Gong et al., 2009).

### Network analysis

Several network topological properties were applied for the weighted anatomical brain network derived from each participant, including small-worldness, efficiency and modularity (Watts and Strogatz, 1998; Latora and Marchiori, 2001; Newman, 2006). The connection weights of all edges (*w*_*ij*_) were normalized by the mean weight of the whole network to keep network cost at the same level for all subjects.

For a weighted network *G* with *N* nodes and *K* edges, the total strength *S* was defined as the mean of all edge weights in the network, $S(G)=\frac{1}{N}\sum_{i\;\neq \;j\;\in \;G}^{N_{M}}w_{ij}$ where *i* and *j* are two distinct nodes in graph *G*. The clustering coefficient (*CC*) of the weighted network *G* quantifies the likelihood whether the neighboring nodes of any network nodes are connected with each other (Onnela et al., 2005), which was defined as: $CC=\frac{1}{N}\sum_{j,k\;\in \;G}{\left(w_{ij}w_{jk}w_{ik}\right)}^{1/3}/(k_{i}^{*}(k_{i}-1)/2)$, where *k*_*i*_ is the number of connected neighbors of node *i*. The weighted characteristic path length *L* of a network is the average minimum connectional weights that link any two nodes of the network. To avoid the issue of disconnected nodes, *L* was measured here by using a “harmonic mean” distance between any pair of nodes such as the reciprocal of the average of the reciprocals (Newman, 2003). A real network would be considered small world if it meets the following criteria: γ = *C*^*real*^_*p*_/*C*^*rand*^_*p*_ > 1 and λ = *L*^*real*^_*p*_/*L*^*rand*^_*p*_ ~ 1 (Watts and Strogatz, 1998), where *C*^*rand*^_*p*_ and *L*^*rand*^_*p*_ are the mean *CC* and *L* of matched random networks that preserve the same number of nodes, edges and degree distribution as the real network (Maslov and Sneppen, 2002). In this study, we generated 1000 matched random networks for each group network.

The global efficiency *E*_*glob*_ of a weighted network *G* is defined as $E_{glob}(G)=\frac{1}{N(N-1)}\sum_{i\;\neq \;j\in \;G}\frac{1}{w_{ij}}$, where *w*_*ij*_ is the smallest connectional weight between node *i* and *j* and *N* is the number of nodes. It characterizes the efficiency of a system transporting information in parallel (Latora and Marchiori, 2003). The local efficiency *E*_*loc*_ of a weighted network *G* is defined as: $E_{loc}(G)=\frac{1}{N}\sum_{i\;\in \;G}E_{glob}(G_{i})$, where *G*_*i*_ denotes the subgraph composed of the nearest neighbors of node *i*. The local efficiency represents the fault tolerance level of the network in response to the removal of a node (Latora and Marchiori, 2003).

The regional global efficiency *E*_*reg*_ of a given node *i* is defined as: $E_{nodal}(i)=\frac{1}{N-1}\sum_{i\;\neq \;j\;\in \;G}\frac{1}{w_{ij}}$, as it measures the average smallest path weight between given node *i* and all other nodes in the network. The node *i* was considered as a hub if its regional global efficiency was at least one standard deviation (*SD*) greater than the mean nodal efficiency of the whole network.

The modularity *Q(p)* for a given partition *p* of a weighted brain structural network is defined as $Q(p)=\sum_{s\;=\;1}^{N_{M}}\left[\frac{w_{s}}{W}-{\left(\frac{W_{s}}{2W}\right)}^{2}\right]$, where *N*_*M*_ is the number of modules, *W* is the total weight of the network, *w*_*s*_ is the sum of the connectional weights between all nodes in module *s*, and *W*_*s*_ is the sum of the nodal weights in module *s*. The modularity index quantifies the difference between the weight of intra-module links of the actual network and that of a random network in which connections are weighted at random (Newman, 2004). The aim of this module identification process is to find a specific partition *p* which yields the largest network modularity, *Q(p)*. Here a modified greedy optimization algorithm (Clauset et al., 2004; Danon et al., 2006) is used to find the modules of the network. The advantage of this modularity optimization method is that it takes into account the heterogeneity of module size observed in real networks (Danon et al., 2006).

We also determined the participation coefficient (PC) for each cortical region in terms of their inter-modular connection density (Guimera and Amaral, 2005; Guimera and Nunes Amaral, 2005; Sales-Pardo et al., 2007). The PC, *P*_*i*_, measures the inter-modular connectivity of node *i*, and is defined as: $P_{i}=1-\sum_{s\;=\;1}^{N_{M}}{\left(\frac{w_{is}}{w_{i}}\right)}^{2}$, where *N*_*M*_ is the number of modules and *w*_*is*_ is inter-modular connectional weight between the node *i* and module *s*. *w*_*i*_ is the total weight of node *i* in the network. The PC of node *i* will be close to 0 if all weights are within its module. The node *i* was considered as an inter-modular hub if its *PC* value was at least one *SD* greater than the mean PC of the whole network.

### Statistical analysis

Between-group differences analysis of all the global network metrics (*S, CC, L, E*_*glob*_, *E*_*loc*_, *E*_*reg*_, *Q*) was performed between adjacent age groups using the General linear model (GLM) with age and gender included as covariates. The nodal properties (*E*_*reg*_, *z, P*) were corrected by false discovery rate at *q* = 0.05 (Genovese et al., 2002; Zeisel et al., 2011).

## Results

### Age-related changes in fiber number and network sparsity

**Figure 2:**
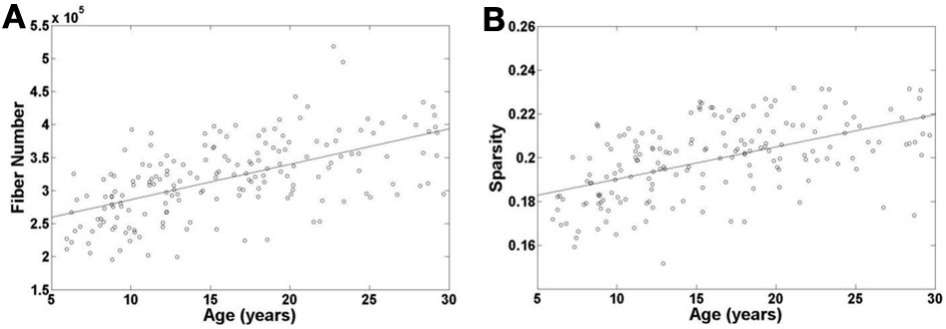
**Age-related changes in fiber number and sparsity of white matter network in all the individual subjects**. Both fiber number and sparsity demonstrate increases with age.

To examine the age effect on the tractography results, we mapped age-related changes in the FN and sparsity of white matter network as shown in Figures 2A,B. We found that age has an incremental effect on both the FN and sparsity, where both increase by a factor of ~1.6 and ~1.2, respectively, from age 6 to 30 years. These increases are presumably due to the known elevations of FA in WM with age. Given the fact that our network edge weighting function depends on FN and FA, it is expected that the connectivity strength of the network would also increase with age.

### Small-world efficiency of developing WM networks

**Figure 3:**
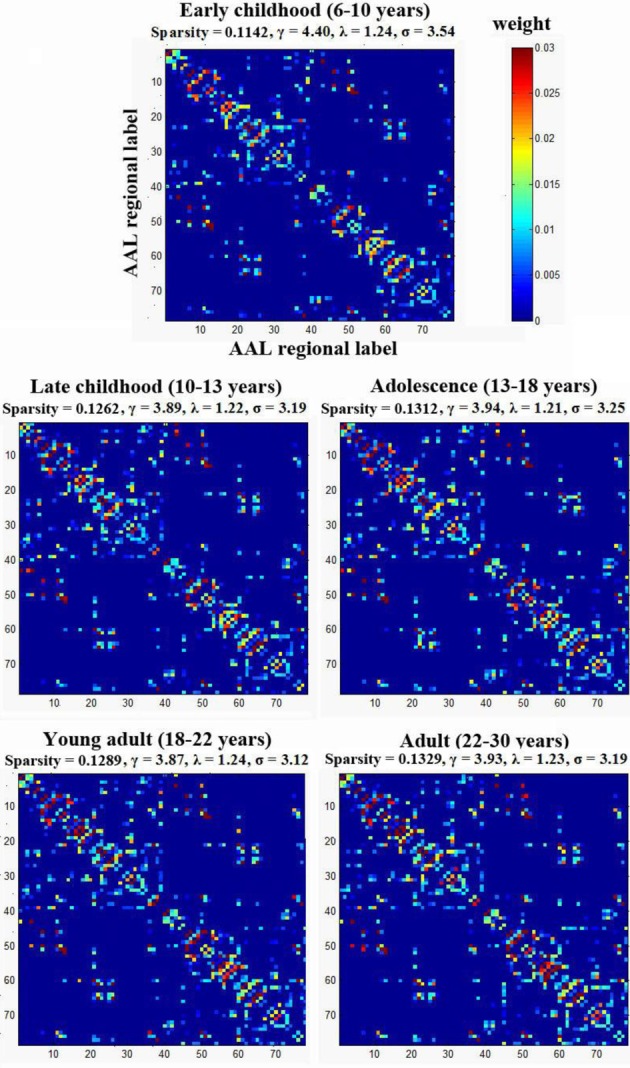
**The small-worldness of the WM networks for the five age groups**. Each group was represented by one weighted backbone network to capture the underlying anatomical connectivity patterns. Compared with their corresponding random networks, all age groups showed strong small-worldness (i.e., σ > > 1). The numbers reflect the structural indices indicated in Table 2.

To examine the small-worldness of the WM networks for all different age groups, using a previously published method by our group (Gong et al., 2009), one weighted backbone network for each age group was generated to capture the underlying anatomical connectivity patterns as shown in Figure 3. Compared with their corresponding 1000 random networks, all five age groups showed strong small-worldness (σ_early childhood_ = 3.54, σ_late childhood_ = 3.19, σ_adolescence_ = 3.25, σ_young adult_ = 3.12, σ_adult_ = 3.19).

### Global network properties and their age-related trajectories

**Figure 4:**
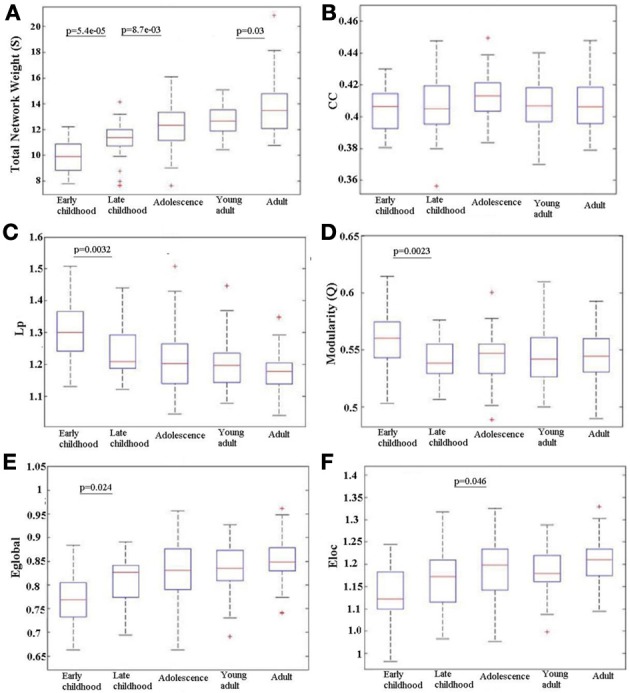
**Age-related changes in different network metrics for the developing WM network from early childhood to adult. (A)** Total network strength (S), **(B)** Clustering coefficient (CC), **(C)** Shortest path length (L_*p*_), **(D)** Modularity (Q), **(E)** Global efficiency (E_glob_), and **(F)** Local efficiency (E_loc_). Significant changes between any adjacent age groups are indicated by their *p* value. An increase with age is observed in S over the full age span. E_glob_ increases only between the two youngest age groups and E_loc_ only between late childhood and adolescence; in both cases, the efficiency values then stay elevated. Reductions are observed in L_*p*_ and Q from early to late childhood that is then maintained low. There is no change in CC between any adjacent age groups. The + signs indicate outliers.

Over all subjects in each age group, the total network weight, CC, Lp, modularity (Q), E_glob_ and E_loc_ was calculated for the WM network and the age-related trajectories are shown in Figure 4. The total network weight displayed significant increases in three of the four developing stages, whereas the other metrics such as L_*p*_, Q, E_glob_, and E_loc_ demonstrated non-linear alteration patterns where most changes happened from young childhood to late childhood that then leveled off. Both L_*p*_ and Q decreased significantly from young childhood to late childhood but stabilized at older ages. Global network efficiency increased significantly from young childhood to late childhood but also stabilized later. Local network efficiency increased significantly from late childhood to adolescence and stabilized afterwards.

### Regional efficiency of the developing WM networks

**Figure 5:**
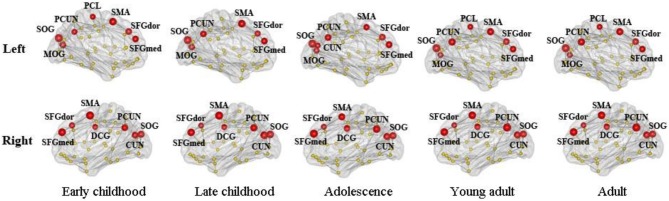
**Global hubs of the developing WM network defined by their nodal efficiency**. Association hub regions such as bilateral PCUN, SFGdor, and SFGmed are consistent over all age groups. Note that all brain images are viewed from the medial side (also for Figures 6, 7).

**Figure 6:**
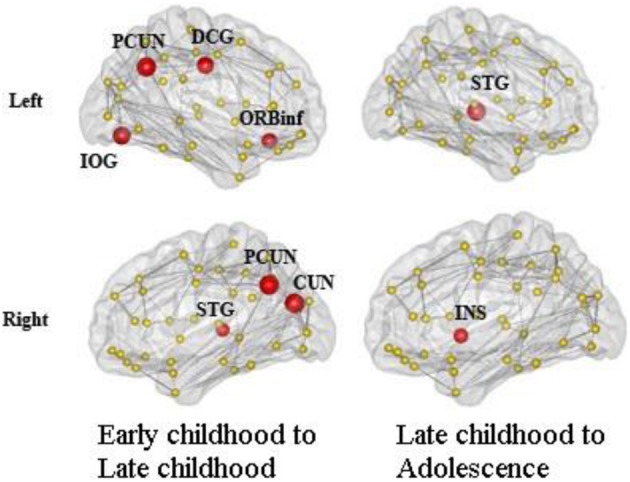
**Regions with significantly increased nodal efficiency from early childhood to late childhood and late childhood to adolescence**. Seven regions had increased nodal efficiency (*P* < 0.05, FDR corrected) from early childhood to late childhood and two regions from late childhood to adolescence. Most regional changes from early to late childhood are in the default-mode system, including bilateral PCUN and left DCG. Left STG and right INS have increased efficiency from late childhood to adolescence.

We found consistent hubs regions, measured here as the AAL areas with highest regional global efficiency, such as bilateral PCUN, SFGdor, and SFGmed, that are shared by all age groups as shown in Figure 5. Comparing the regional efficiency changes from group to group in these hubs, seven regions had increased nodal efficiency (*P* < 0.05, FDR corrected) from early childhood to late childhood and two regions from late childhood to adolescence (Figure 6). Most regional changes from early to late childhood are in the default-mode system, including bilateral PCUN and left DCG. Left STG and right INS were found to have increased efficiency from late childhood to adolescence.

### Modular organization and connector hubs of the developing WM networks

**Figure 7:**
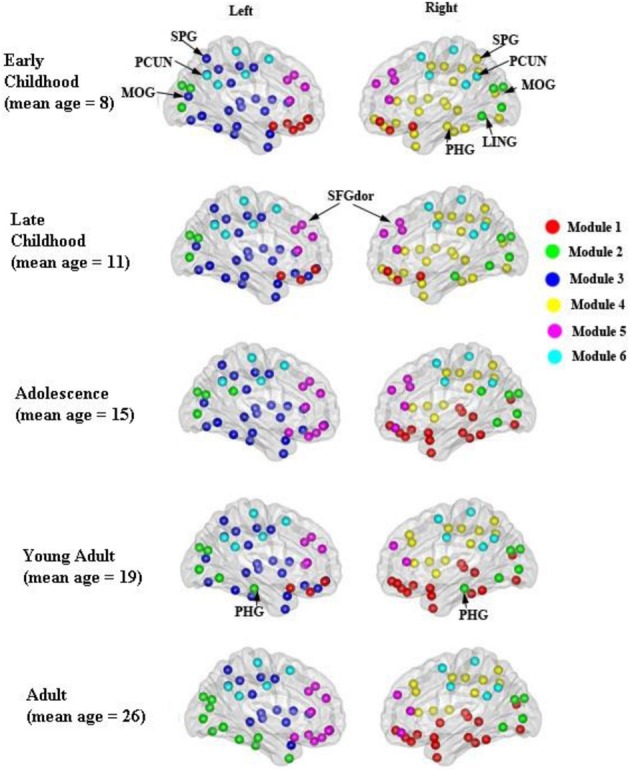
**Modular organization of the developing WM networks**. Six **modules (1–6)** were detected in all age groups and are represented by red, green, purple, yellow, pink, and blue colors. See Table 3 for a detailed list of modular regions. Module 1 was mostly composed of bilateral orbitofrontal regions (ORBsup, ORBsupmed, ORBmid, REC) in early and late childhood and becomes more lateralized from adolescence onwards. Module 2 consists of mostly occipital regions (SOG, CAL, CUN) bilaterally. Lateralized modules 3 (left hemisphere) and 4 (right hemisphere) consist of regions mostly across frontal, parietal and temporal lobes within each hemisphere. Modules 5 and 6 are two of the most consistent modules during development that include mostly bilateral frontal (SFG, MFG) and posterior parietal (PCUN, DCG, SMA) regions, respectively.

The modular organization of the developing structural brain networks for the five different age groups is shown in Table 3 and Figure 7. Six modules (1–6) were detected in all age groups indicating strong stability (Greicius et al., 2003) in the modularity of the developing brain network. Despite decreased modularity from early to late childhood, the modular structures of both groups were almost identical. Module 1 was mostly composed of bilateral orbitofrontal regions (ORBsup, ORBsupmed, ORBmid, REC) in early and late childhood that becomes more lateralized in adolescence. Right orbitofrontal regions become connected with right temporal and occipital regions that resembles the ventral visual system (Grill-Spector et al., 2008) and left orbitofrontal regions become part of lateral frontal system. Module 2 consists of mostly occipital regions (SOG, CAL, CUN) throughout the youngest age groups except at adulthood when the left occipital regions become part of left ventral visual system (Grill-Spector et al., 2008). Lateralized modules 3 (left hemisphere) and 4 (right hemisphere) consist of regions mostly across frontal, parietal and temporal lobes from each hemisphere from early to late childhood. However, module 4 is pruned to a mainly frontal-parietal system from adolescence onwards and module 3 doesn't reach a similar outcome until adulthood. Modules 5 and 6 are two of the most consistent modules during development and include mostly bilateral frontal (SFG, MFG) and posterior parietal (PCUN, DCG, SMA) regions, respectively.

**Table 3:** **Cortical regions in each module of developing white matter network**.

**Module**	**Early childhood**	**Late childhood**	**Adolescence**	**Young adult**	**Adult**
1	ORBsupmed.L	ORBsupmed.L	ORBsup.R	ORBsupmed.L	ORBsup.R
	ORBsupmed.R	ORBsupmed.R	ORBmid.R	ORBsup.R	ORBmid.R
	ORBsup.L	REC.L REC.R	ORBinf.R	ORBsupmed.R	ORBinf.R
	ORBmid.L	OLF.L	TPOsup.R	ORBmid.R	TPOsup.R
	ORBinf.L	OLF.R	TPOmid.R	ORBinf.R	TPOmid.R
	REC.L		REC.R OLF.R	TPOsup.R	MOG.R IOG.R
	REC.R		IOG.R FFG.R	TPOmid.R	FFG.R HES.R
	OLF.L		HES.R STG.R	REC.L REC.R	STG.R MTG.R
	OLF.R		MTG.R ITG.R	OLF.L OLF.R	ITG.R PHG.R
			MOG.R PHG.R	IOG.R FFG.R	REC.R OLF.R
				HES.R STG.R	INS.R
				MTG.R ITG.R	
				MOG.R	
2	SOG.L SOG.R	SOG.L SOG.R	SOG.L CAL.L	SOG.L SOG.R	TPOmid.L
	CAL.L CAL.R	CAL.L CAL.R	CUN.L PCG.L	CAL.L CAL.R	SOG.L SOG.R
	CUN.L CUN.R	CUN.L CUN.R	SOG.R CAL.R	CUN.L CUN.R	MOG.L IOG.L
	LING.R	LING.R PHG.R	CUN.R LING.R	LING.L LING.R	CAL.L CAL.R
			PCG.R	PHG.L PHG.R	CUN.L CUN.R
					LING.L LING.R
					FFG.L ITG.L
					PHG.L
3	IFGoperc.L	ORBsup.L	ORBmid.L	ORBsup.L	IFGoperc.L
	IFGtriang.L	ORBmid.L	ORBinf.L	ORBmid.L	IFGtriang.L
	TPOsup.L	ORBinf.L	IFGoperc.L	ORBinf.L	TPOsup.L
	TPOmid.L	IFGoperc.L	IFGtriang.L	IFGoperc.L	PreCG.L ROL.L
	PreCG.L ROL.L	IFGtriang.L	TPOsup.L	TPOsup.L	PoCG.L SPG.L
	PoCG.L SPG.L	TPOsup.L	TPOmid.L	TPOmid.L INS.L	IPL.L SMG.L
	IPL.L SMG.L	TPOmid.L	PreCG.L ROL.L	PreCG.L ROL.L	ANG.L HES.L
	ANG.L MOG.L	PreCG.L ROL.L	PoCG.L SPG.L	PoCG.L SPG.L	STG.L MTG.L
	IOG.L LING.L	PoCG.L SPG.L	IPL.L SMG.L	IPL.L SMG.L	INS.L
	FFG.L HES.L	IPL.L SMG.L	ANG.L MOG.L	ANG.L MOG.L	
	STG.L MTG.L	ANG.L MOG.L	IOG.L LING.L	IOG.L FFG.L	
	ITG.L PHG.L	IOG.L LING.L	FFG.L HES.L	HES.L STG.L	
	INS.L	FFG.L HES.L	STG.L MTG.L	MTG.L ITG.L	
		STG.L MTG.L	ITG.L PHG.L		
		ITG.L PHG.L	INS.L		
		INS.L			
4	ORBsup.R	ORBsup.R	IFGoperc.R	SFGdor.R	SFGdor.R
	ORBmid.R	ORBmid.R	IFGtriang.R	IFGoperc.R	IFGoperc.R
	ORBinf.R	ORBinf.R	PreCG.R	IFGtriang.R	IFGtriang.R
	IFGoperc.R	IFGoperc.R	PoCG.R	PreCG.R	PreCG.R
	IFGtriang.R	IFGtriang.R	ROL.R	PoCG.R	PoCG.R
	TPOsup.R	TPOsup.R	SPG.R	MFG.R	MFG.R
	TPOmid.R	TPOmid.R	IPL.R	ROL.R	ROL.R
	PHG.R INS.R	INS.R PreCG.R	SMG.R	SPG.R	SPG.R
	PreCG.R ROL.R	ROL.R PoCG.R	ANG.R	IPL.R	IPL.R
	PoCG.R SPG.R	SPG.R IPL.R	INS.R	SMG.R	SMG.R
	IPL.R SMG.R	SMG.R ANG.R		ANG.R	ANG.R
	ANG.R MOG.R	MOG.R IOG.R		INS.R	
	IOG.R FFG.R	FFG.R HES.R			
	HES.R STG.R	STG.R MTG.R			
	MTG.R ITG.R	ITG.R			
5	SFGdor.L	SFGdor.L	SFGdor.L	SFGdor.L	SFGdor.L
	SFGdor.R	SFGdor.R	SFGmed.L	SFGmed.L	SFGmed.L
	SFGmed.L	ORBsup.L	SFGmed.R	SFGmed.R	SFGmed.R
	SFGmed.R	ORBsupmed.L	IFGtriang.L	MFG.L	ORBsup.L
	ACG.L	ORBsupmed.R	MFG.L	IFGtriang.L	ORBsupmed.R
	ACG.R	SFGmed.L	ACG.L	ACG.L	ORBsupmed.L
	MFG.L	SFGmed.R	ACG.R	ACG.R	ORBmid.L
	MFG.R	REC.L OLF.L			ORBinf.L
		MFG.L MFG.R			MFG.L
		ACG.L ACG.R			ACG.L ACG.R
					REC.L OLF.L
6	PCUN.L PCUN.R	PCUN.L PCUN.R	PCUN.L PCUN.R	PCL.L PCUN.L	PCUN.L PCUN.R
	DCG.L DCG.R	DCG.L DCG.R	DCG.L DCG.R	PCUN.R DCG.L	DCG.L DCG.R
	PCG.L PCG.R	PCG.L PCG.R	PCL.L PCL.R	DCG.R PCG.L	PCG.L PCG.R
	PCL.L PCL.R	PCL.L PCL.R	SMA.L SMA.R	PCG.R PCL.R	PCL.L PCL.R
	SMA.L SMA.R	SMA.L SMA.R		SMA.L SMA.R	SMA.L

**Figure 8:**
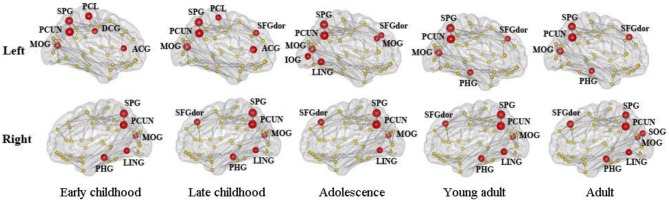
**The distribution of inter-modular hubs based on participation coefficient (PC) of each region for different age groups**. They were mostly located within posterior cortex, including bilateral PCUN, SPL, and MOG. Large frontal hubs such as bilateral SFGdor appeared in late childhood and remained significant afterwards.

The distribution of inter-modular hubs based on PC of each region for different age groups was very consistent (Figure 8). They were mostly located within posterior cortex, including bilateral PCUN, SPL, and MOG. Large frontal hubs such as bilateral SFGdor appeared in late childhood and remained significant afterwards.

To ensure the change of modular organization didn't result from the different sparsity of the five age groups, an additional analysis was performed where we normalized all group networks sparsity to the lowest sparsity at 0.1142 (early childhood) as weaker connections were removed from the other networks with higher sparsity (late childhood, 0.1262, adolescence, 0.1312, young adult, 0.1289, adult, 0.1329) and re-examined the modularity of the networks. We found extremely consistent modularity (0.56 previous vs. 0.56 with normalized sparsity in late childhood, 0.56 vs. 0.56 in adolescence, 0.55 vs. 0.56 in young adults, and 0.58 vs. 0.56 in adults) and modular organization compared with our original networks. Thus, we could presume that changes in the backbone network and its modular organization were not due to different matrix densities.

## Discussion

The present study utilized DTI tractography and network theory to characterize changes to the global structural WM network with age from early childhood to adulthood. Our main results are demonstrations of (1) a non-linear age effect on most network topological properties of brain WM network in development where most changes happen at late childhood stage (10–13 years), such as increased global network efficiency and decreased modularity, suggesting a shift of organization toward a more randomized configuration, (2) consistent hubs involving several major functional systems across all age groups and significant nodal changes only happening from early childhood to adolescence, (3) anatomically localized modules in the development of brain WM network, and (4) key connector hubs during development of the WM network.

First, using graph theoretical analysis, small-world network architecture was demonstrated in the WM networks of all age groups. During the last decade, graph theoretical analysis has been widely applied to both the functional (Stam, 2004; Bassett et al., 2006; Achard and Bullmore, 2007) and anatomical (He et al., 2007; Hagmann et al., 2008; Gong et al., 2009) brain networks and one common finding is the existence of “small-worldness” in all types of network, as defined by high CC and low characteristic path length. Recent structural brain network studies have also revealed that small-world topology and modular organization are established during early brain development (<2 years) to support rapid synchronization and information transfer with minimal rewiring cost (Fan et al., 2011; Yap et al., 2011). Thus, our results are in agreement with previous findings that the WM network maintains small-world efficiency at all stages of development.

Total network weight shows increases in three of the four developing stages, although the greatest change is between the two youngest groups pre-adolescence. Our finding is consistent with a previous WM network study that reported a significant increase in the average node strength in a group of subjects aged from 18 months to 18 years where it was suggested that increased network weight indicates increased nodal strength and greater physiological efficacy, particularly of long pathways (Hagmann et al., 2010). A functional network study has also reported increased functional integration due to a decrease of average path length during the same period and suggested it was related to increased axonal diameter and myelin thickness of long association fiber tracts (Supekar et al., 2009). We also found significant age-related decreases in the shortest path length and modularity and increase in the global efficiency of the developing WM network from early childhood to late childhood indicating greater integration among distant brain regions and a shift of topological organization to a more randomized configuration. Previous WM network (Hagmann et al., 2010) and cortical thickness network (Khundrakpam et al., 2013) studies of brain development also demonstrated a similar pattern of network metrics evolution between age 2 and 18 years and between 5 and 18 years, respectively. However, the WM network study had applied a linear fit for all the network metrics vs. age even though network metrics such as efficiency and clustering seemed to have leveled off after late childhood in their paper (Hagmann et al., 2010). Using a similar approach to ours, (Khundrakpam et al., 2013) demonstrated a leveling off of various cortical thickness network metrics after the early adolescence stage.

Consistent global hub regions, indicated by higher regional efficiency, are observed across all age groups. Hub regions are predominately association cortices that receive convergent inputs from multiple cortical regions. Regions such as SFG and PCUN have been constantly identified as the hub regions in both structural (He et al., 2007; Gong et al., 2009) and functional brain networks (Achard and Bullmore, 2007). A recent structural brain network study also identified them as the hub regions from age 2 years suggesting that they are established at a very early age (Hagmann et al., 2010). We also found that the regions with the most age-related increases in efficiency are in the default-mode system, including bilateral PCUN and left DCG. A functional brain network study has reported a less well-developed default mode network connectivity in early childhood compared with adults, especially within posterior regions such as PCUN (Fair et al., 2008). However, evidence from structural covariance network analysis has demonstrated significant pruning in the default mode system from early childhood to late childhood (Zielinski et al., 2010). Thus, we could speculate that nodal efficiency of default mode regions might plateau by late childhood.

In this study, a stable and functionally/anatomically related modular organization was demonstrated in the developing WM network. Six modules comprising regions with known functions or connections were identified in the developing WM network. Modules 1, 2, 5 and 6 were mostly composed of orbitofrontal, occipital, frontal, and posterior parietal regions that could correspond to sensory integration, visual, executive function, and default mode network, respectively, (Duncan and Owen, 2000; Raichle et al., 2001; Kringelbach, 2005). Modular network analysis has provided rich quantitative insights into the organization of complex brain networks. Studies in mammalian anatomical brain networks have revealed clusters that overlap with many known brain functions (Hilgetag et al., 2000; Zhou et al., 2006). Previous neuroimaging studies have also demonstrated anatomically- and functionally-related modules in the human brain structural network using diffusion spectrum imaging (Hagmann et al., 2008) and the functional network using resting-state functional MRI (Salvador et al., 2005; Ferrarini et al., 2009; He et al., 2009; Meunier et al., 2009; Valencia et al., 2009). Also, network modules identified by cortical thickness network analysis are comprised of brain regions known to subserve distinct brain functions such as executive function, vision, and default mode network (Chen et al., 2008, 2011). Two recent DTI studies also revealed non-random and dynamic modular organization of structural brain network in the first 2 years of brain development (Fan et al., 2011; Yap et al., 2011). Two lateralized modules (3 and 4) that correspond to the frontal-parietal network were also observed in the developing WM network. The adult human brain exhibits distinct hemispheric asymmetries in both structure and function. These asymmetries are thought to originate from evolutionary, developmental, hereditary, experiential, and pathological factors (Toga and Thompson, 2003). Thus, we could speculate that the lateralized network modules might result from the functional and structural hemispheric asymmetries.

Taken together, our results suggest an efficient modular organization in the WM network from early childhood and are consistent with modular behavior reported in previous structural and functional brain network studies and more importantly, a lateralized developmental pattern in some of the modules. The inter-modular hubs are the main connectors between modules and their existence in frontal and posterior cortex in the developing brain are consistent with previous WM network (Yap et al., 2011) and cortical thickness network (Khundrakpam et al., 2013) analysis. Resting state functional networks have also reported a high density of strong functional connections in posterior cortex (Achard et al., 2006). Thus, we could speculate that the inter-modular hubs uncovered in this study are well-established at childhood and are responsible for the connections between different functional systems of the developing brain.

A few methodological issues need to be addressed. Two drawbacks of our study include the acquisition of DTI data with six diffusion directions at low *b* values of 1000 s/mm^2^ and the use of deterministic tractography which will give errors in such an unsupervised tractography method given abrupt terminations at low FA crossing fiber regions or erroneous connections due to errors in the primary eigenvector direction. Multiple gradient directions can reduce the uncertainty of the primary eigenvector direction and limit potential bias as a function of tract orientation, both concerns for deterministic tractography of WM tracts (Landman et al., 2007). However, a recent study from our group has demonstrated six-direction data can also provide average diffusion measures like FA over a specific tract with comparable robustness to 30- or 60-direction data and yield appropriate parameter values for many major WM tracts (Lebel et al., 2012), which is encouraging as our edge weights were calculated based on the average FA of all voxels over the whole tract connecting two nodes. However, this does not overcome potential false positive connections or missed connections from the deterministic tractography algorithm. We attempted to minimize the former by invoking a minimum FN between regions but an incorrect connection that is consistent among the subjects within a group would still be included in the network analysis. DTI data with more than six directions also permit other advantages such as alternative analysis methods (e.g., probabilistic tractography) (Dennis et al., 2013). Higher *b* values than typically acquired are also advantageous for resolving crossing fibers and increasing the accuracy of tractography derived connections (Tournier et al., 2008). Another limitation of the study is that the age ranges of the groups covered a 3.1 to 4.7 year age range for the four youngest groups. In this study, a general linear model was applied to remove those age effects within all groups before performing the between-group comparison. In future study, smaller age ranges within groups may provide more specific indices of timing for the WM network maturation. Third, a FN threshold of 10 was applied to minimize the inclusion of random connections between two cortical regions. Currently, there are no standard approaches in determining the threshold value for the number of connecting fibers between regions as small thresholds such as 3 streamlines (Shu et al., 2011) produced networks with large sparsity with many spurious connections. Thus, our choice of higher threshold reduces, but does not eliminate, the risk of false-positive connections due to noise or the limitations in deterministic tractography. Recently, a threshold of 10 connecting streamlines or more was also applied in a brain network study (van den Heuvel et al., 2012) in which they considered that edges comprising fewer than 10 streamlines were potentially spurious and were deleted from the connection matrix. To examine the influence of the threshold, we tested a range of thresholds from 3 to 10 fibers and results including all network parameters are shown in Table A1. Although the network efficiency decreased as the sparsity decreased, the small worldness of the network remained intact. Most importantly, the group differences among adjacent age groups also remained consistent across all applied thresholds which indicates that the network comparison results are not sensitive to the threshold choices. Cortical regions in our study are defined by an a priori volumetric template (AAL) that was employed to automatically parcellate the entire cerebral cortex into different regions. Different templates used in various studies might cause discrepancy in the specific results, though the main trend of the network properties is expected to remain intact.

Various weighting functions for cortical-cortical connections have been applied in previous brain network analyses of brain development including 1/mean diffusivity (Hagmann et al., 2010) and proportional FN (Dennis et al., 2013), whereas we used the product of tract FA (known to increase exponentially with development over this age range but at unique rates per tract—Lebel et al., 2008) and AAL regional volume-normalized FN that has been used by others in studies of Alzheimer's Disease and aging (Lo et al., 2010; Brown et al., 2011). Other diffusion indices such as mean diffusivity (MD), axial diffusivity (AD) or radial diffusivity (RD) could have been examined instead of FA as a basis of “weighting” the network connections. However, to our best knowledge, while a few studies have applied MD as an edge weighting function (Hagmann et al., 2010; Li et al., 2012), none have used AD or RD. While changes in MD and FA for the WM typically occur together during maturation, with *MD* values decreasing and *FA* values increasing, the processes by which the two parameters change are theoretically different (Schmithorst et al., 2002; Huppi and Dubois, 2006) and they do not change at the same rate (Lebel et al., 2008). Axial and RD, under certain circumstances, may be more specific to underlying biological processes, such as myelin and axonal changes (Song et al., 2002). A recent study has demonstrated changes of FA in corticospinal tract and anterior corona radiata during development (2 to 40 years) that were attributed to the different rate changes in AD and RD (Faria et al., 2010). Thus, one would expect different WM network organization if using different weighting functions. Therefore, future studies could consider using multiple diffusion tensor measures such as FA, MD, AD and RD.

In conclusion, a graph theoretical approach was used to demonstrate age-related alterations in the large scale network properties of the developing WM network from early childhood (6 years) to adulthood (30 years). It was shown that increased network weight signifies a reshaping of the WM network from early childhood to late childhood with increased integration and decreased segregation. These findings are compatible with the notion that structural and functional brain networks become stable after late childhood. Our results also have implications for understanding how the modular organizational alterations in the large-scale structural brain networks underlie maturation of cognitive function in brain development. This study may pave the way for developing novel methods for analyzing disrupted brain connectivity in neurodevelopmental disorders.

### Conflict of interest statement

The authors declare that the research was conducted in the absence of any commercial or financial relationships that could be construed as a potential conflict of interest.

## Figures and Tables

**Table A1:**
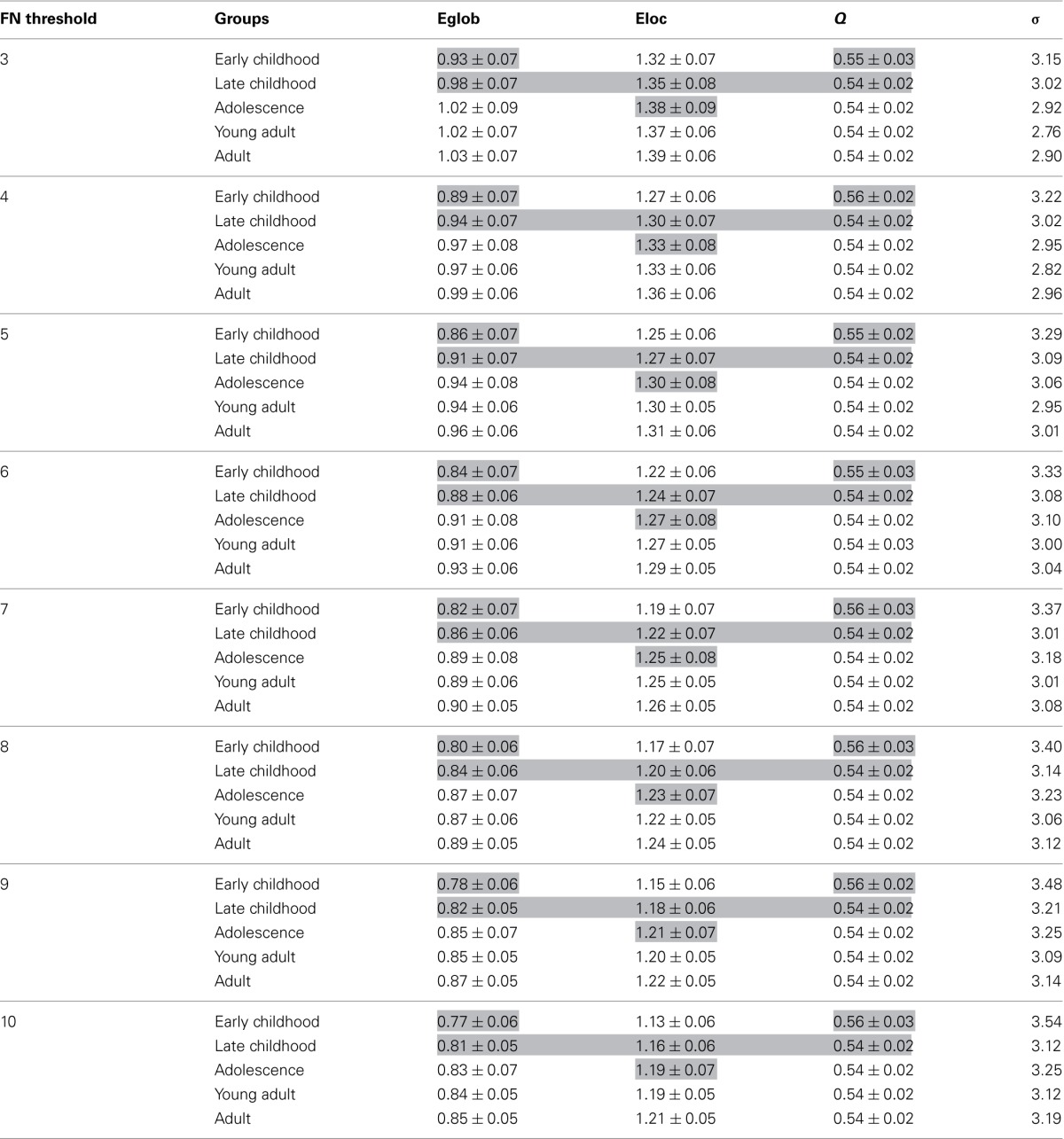
**White matter network parameters derived from different fiber-number node-to-node connection thresholds in all age groups**.

Adjacent groups show significant differences or trend in network properties are highlighted in shaded cells. FN: fiber number, Eglob: global efficiency, Eloc: local efficiency, Q: modularity, σ: small worldness.
